# Supplementary material for: Encrypted federated learning for secure decentralized collaboration in cancer image analysis
Source: Med Image Anal. 2024 Feb;92:103059. doi: 10.1016/j.media.2023.103059 (PMC10804934; doi:10.1016/j.media.2023.103059)
Supplement: Supplementary file 1 [file mmc1.docx]

**Supplemental material**

**S1. Further details on the utilized cohorts**

Tables S1 and S2 show further details on the radiology and pathology datasets utilized in this study.

| Data source | Number of examinations | Allocation to federated learning center number |
| --- | --- | --- |
| BraTS center 1 | 129 | 1 |
| BraTS center 2 | 14 | 2 |
| BraTS center 3 | 5 | 2 |
| BraTS center 4 | 9 | 2 |
| BraTS center 5 | 22 | 2 |
| BraTS center 6 | 34 | 3 |
| BraTS center 7 | 12 | 3 |
| BraTS center 8 | 8 | 3 |
| BraTS center 9 | 4 | 3 |
| BraTS center 10 | 8 | 4 |
| BraTS center 11 | 6 | 4 |
| BraTS center 12 | 9 | 4 |
| BraTS center 13 | 27 | 4 |
| BraTS center 14 | 3 | 4 |
| BraTS center 15 | 11 | 4 |
| BraTS center 16 | 34 | 5 |
| BraTS center 17 | 6 | 5 |
| BraTS unknown group | 28 | 5 |
| Sum | 369 |  |

**Table S1**. Allocation of patients in the BraTS collective to five data clusters used in the federated learning setup simulating the situation in which five regional hospitals’ image databases contain a multitude of examinations from different scanners.

|  | **TCGA** | **DACHS** | **Epi700** | **QUASAR** | **YCR-BCIP** |
| --- | --- | --- | --- | --- | --- |
| Number of patients | 632 | 2448 | 661 | 2190 | 889 |
| Age (median ± IQR) | 68 ± 18 | 69 ± 14 | 72 ± 14 | 63 ± 12 | 71 ± 15 |
| Gender: Male | 322 (50.9%) | 1436 (58.7%) | 358 (54.2%) | 1334 (60.9%) | 494 (55.6%) |
| Gender: Female | 292 (46.2%) | 1012 (41.3%) | 303 (45.8%) | 848 (38.7%) | 395 (44.4%) |
| Gender: Unknown | 18 (2.85%) | 0 (0%) | 0 (0%) | 8 (0.4%) | 0 (0%) |
| MSS/pMMR | 392 (62%) | 1836 (75%) | 471 (71.3%) | 1529 (69.8%) | 760 (85.5%) |
| MSI/dMMR | 65 (10.3%) | 210 (8.6%) | 136 (20.6%) | 246 (11.2%) | 129 (14.5%) |
| Unknown MSI status | 175 (27.7%) | 402 (16.4%) | 54 (8.1%) | 415 (19%) | 0 (0%) |
| Stage 1 | 76 (12%) | 485 (19.8%) | 0 (0%) | 5 (0.2%) | 169 (19%) |
| Stage 2 | 166 (26.3%) | 801 (32.7%) | 394 (59.6%) | 53 (2.4%) | 317 (35.7%) |
| Stage 3 | 140 (22.2%) | 822 (33.6%) | 267 (40.4%) | 1653 (75.5%) | 370 (41.6%) |
| Stage 4 | 63 (10%) | 337 (13.8%) | 0 (0%) | 268 (12.2%) | 33 (3.7%) |
| Stage unknown | 187 (29.5%) | 3 (0.1%) | 0 (0%) | 211 (9.7%) | 0 (0%) |
| Left-sided CRC | 248 (39.2%) | 1607 (65.6%) | 280 (42.3%) | 1158 (52.9%) | 487 (54.8%) |
| Right-sided CRC | 176 (27.8%) | 819 (33.5%) | 375 (56.7%) | 754 (34.4%) | 332 (37.3%) |
| Unknown side | 209 (33%) | 22 (0.9%) | 6 (1%) | 278 (12.7%) | 70 (7.9%) |

**Table S2.** Clinico-pathological features of histopathological cohorts in our study. Abbreviations: colorectal cancer (CRC), interquartile range (IQR), microsatellite instability (MSI), microsatellite stability (MSS). Definitions: Right-sided CRC from cecum to transverse colon.

**S2. Ablation study**

Figures S1 and S2 show an ablation study on histopathological data, where the proposed SHEFL method is performed for varying numbers of local sites and their samples and different architectures. In all cases, we observed the superiority of SHEFL as compared to local models, while no difference was observed to conventional FL.

**
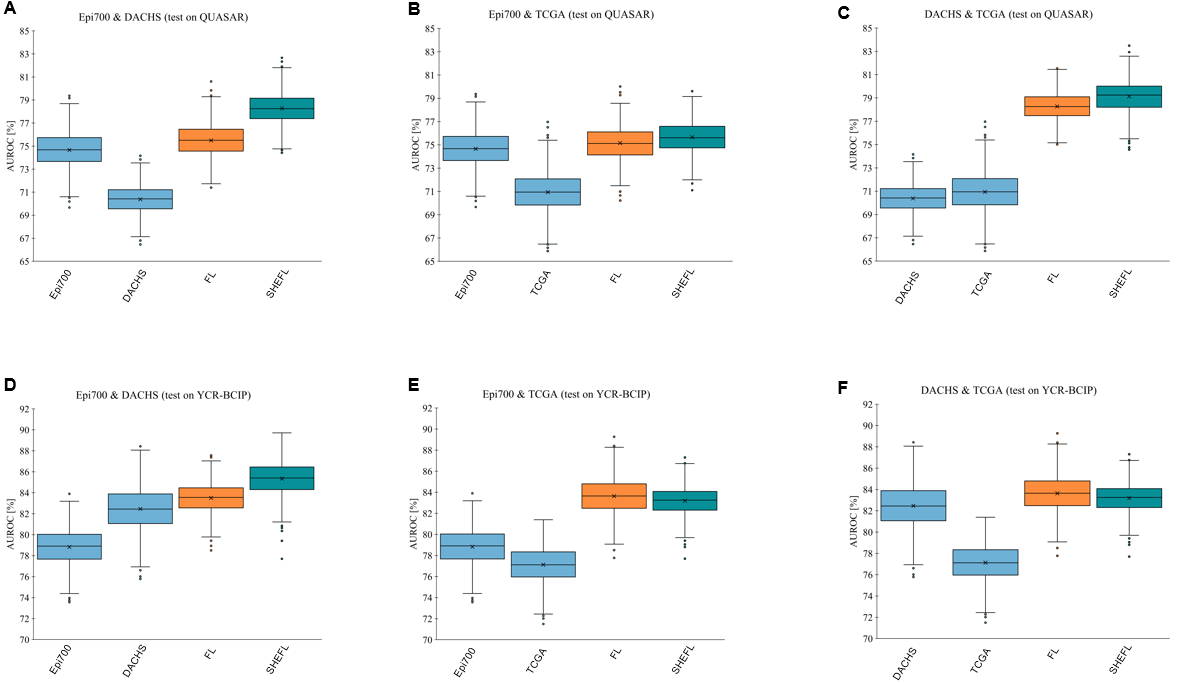
**

**Figure S1.** Results of ablation study for histopathology utilizing different local sites. Training neural networks on single-site datasets resulted in inferior performance as compared to FL and SHEFL. A neural network was trained to detect MSI on data from Epi700 and DACHS **(A,D)**, Epi700 and TCGA **(B,E)**, and DACHS and TCGA **(C,F)** cohorts locally, using FL, and SHEFL. The resulting networks were then tested on the QUASAR **(A–C)** and the YCR-BCIP **(D–F)** cohorts demonstrating superior performance of FL and SHEFL.

**
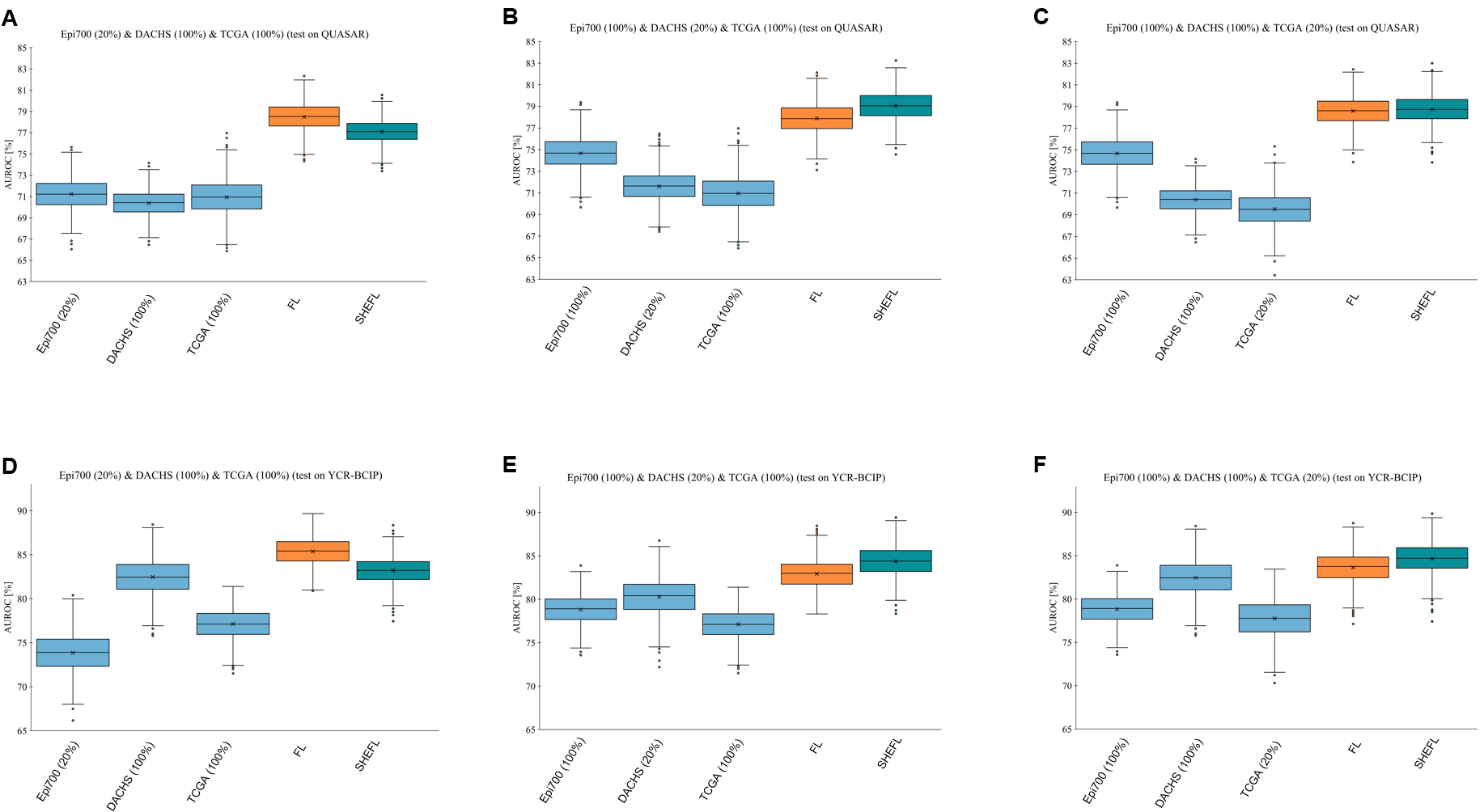
**

**Figure S2.** Results of ablation study for histopathology utilizing different sample sizes. Training neural networks on single-site datasets resulted in inferior performance as compared to FL and SHEFL. A neural network was trained to detect MSI on data from Epi700 (20% of the samples at random), DACHS (full dataset), and TCGA (full dataset) **(A,D)**, Epi700 (full dataset), DACHS (20% of the samples at random), and TCGA (full dataset) **(B,E)**, and Epi700 (full dataset), DACHS (full dataset), and TCGA (20% of the samples at random) **(C,F)** cohorts locally, using FL, and SHEFL. The resulting networks were then tested on the QUASAR **(A–C)** and the YCR-BCIP **(D–F)** cohorts demonstrating superior performance of FL and SHEFL.
